# Monocyte Scintigraphy in Rheumatoid Arthritis: The Dynamics of Monocyte Migration in Immune-Mediated Inflammatory Disease

**DOI:** 10.1371/journal.pone.0007865

**Published:** 2009-11-17

**Authors:** Rogier M. Thurlings, Carla A. Wijbrandts, Roelof J. Bennink, Serge E. Dohmen, Carlijn Voermans, Diana Wouters, Elena S. Izmailova, Danielle M. Gerlag, Berthe L. F. van Eck-Smit, Paul P. Tak

**Affiliations:** 1 Department of Clinical Immunology and Rheumatology, Academic Medical Center, University of Amsterdam, Amsterdam, Noord Holland, The Netherlands; 2 Department of Nuclear Medicine, Academic Medical Center, University of Amsterdam, Amsterdam, Noord Holland, The Netherlands; 3 Landsteiner Laboratory, Department of Experimental Immunohematology, Sanquin Research, Amsterdam, Noord Holland, The Netherlands; 4 Department of Immunopathology, Sanquin Research, Amsterdam, Noord Holland, The Netherlands; 5 Millennium Pharmaceuticals, Inc, Department of Research and Development, Cambridge, Massachusetts, United States of America; Genentech, United States of America

## Abstract

**Background:**

Macrophages are principal drivers of synovial inflammation in rheumatoid arthritis (RA), a prototype immune-mediated inflammatory disease. Conceivably, synovial macrophages are continuously replaced by circulating monocytes in RA. Animal studies from the 1960s suggested that macrophage replacement by monocytes is a slow process in chronic inflammatory lesions. Translation of these data into the human condition has been hampered by the lack of available techniques to analyze monocyte migration in man.

**Methods/Principal Findings:**

We developed a technique that enabled us to analyze the migration of labelled autologous monocytes in RA patients using single photon emission computer tomography (SPECT). We isolated CD14+ monocytes by CliniMACS in 8 patients and labeled these with technetium-99m (^99m^Tc-HMPAO). Monocytes were re-infused into the same patient. Using SPECT we calculated that a very small but specific fraction of 3.4×10^−3^ (0.95−5.1×10^−3^) % of re-infused monocytes migrated to the inflamed joints, being detectable within one hour after re-infusion.

**Conclusions/Significance:**

The results indicate monocytes migrate continuously into the inflamed synovial tissue of RA patients, but at a slow macrophage-replacement rate. This suggests that the rapid decrease in synovial macrophages that occurs after antirheumatic treatment might rather be explained by an alteration in macrophage retention than in monocyte influx and that RA might be particularly sensitive to treatments targeting inflammatory cell retention.

## Introduction

Macrophages in the inflamed synovial tissue of rheumatoid arthritis (RA) patients play a central role in the sustenance of synovial inflammation and promotion of tissue destruction [1]–[3]. Conceivably they are continuously replaced by circulating monocytes [4]. The dynamics of this replacement is a matter of controversy. Data on the effects of anti-rheumatic treatments suggest this might be a highly dynamic process [5]–[11], while animal studies from the 1960s suggested it might occur at a slow rate [12]–[15].

Newly developed imaging techniques, such as Single Photon emission Computed Tomography (SPECT), Positron Emission Tomography (PET) and more recently bioluminescence and fluorescence reflectance imaging, offer the possibility to portray the in vivo dynamics of cell migration in patients [16]. The application of these imaging modalities to analyze the behavior of monocytes is hampered by the relative scarcity of these cells in the peripheral blood and the technical difficulties of specific cell isolation at the GMP level and efficient labeling to result in an adequate detection signal. These problems might be addressed by the combination of scintigraphic imaging with sophisticated cell isolation procedures, such as immunomagnetic cell selection [17].

We recently developed a procedure using a combination of immunomagnetic cell selection with CD14 coated beads and an improved labeling procedure with technetium-99m (^99m^Tc)- hexamethylpropylene-amino-oxime (HMPAO) and SPECT to visualize the migratory behavior of autologous monocytes [18], [19]. We applied this method in patients with active RA to test the hypothesis that synovial inflammation is maintained by a continuous influx of monocytes into the synovial compartment and to analyze the dynamics of such influx.

## Results

Eight RA patients (4 male and 4 female) were included into the study. The median age of the patients was 52 years (range 39 to 59 years) and the mean disease duration was 19 (range 10–38) years. Erosions were present in all patients. Two patients had nodular disease. Four patients were seropositive for IgM rheumatoid factor. The mean (±SD) disease activity score evaluated in 28 joints (DAS28) at screening was 5.8±0.8. All patients were treated with stable dosages of methotrexate.

Applying immunomagnetic cell selection with CD14 labeled beads, on average 19.9×10^6^ (10.4−36.9×10^6^) monocytes were isolated, with a mean recovery of 40.8% (24–69%) CD14 positive cells. This resulted in a cell suspension with a purity of 90.4% (79–96%) CD14 positive cells as determined by FACS analysis. Labeling with ^99m^Tc-HMPAO resulted in a mean radioactivity of 211 (43–393) MBq. Having shown that CD62L expression on monocytes did not change after the bead isolation procedure and that ^99m^Tc-HMPAO labeling did not affect the monocyte migratory capacity in vitro (unpublished observations), we decided to re-infuse labeled monocytes in RA patients. Re-infusion was well tolerated in all patients. No signs of increased complement activation could be demonstrated one hour after re-infusion of radioactively labeled monocytes: C3b/c (mean±SD): 26.4±13.5 and C4b/c 8.3±1.5 before treatment versus 26.0±12.3 and 16.2±10.0 1 hour after re-infusion, respectively).

**Figure 1:**
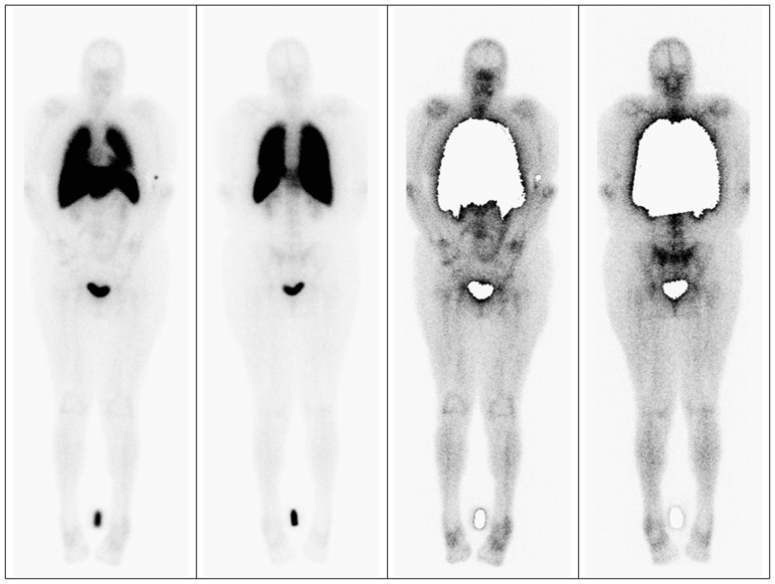
Scintigraphic images of labeled autologous monocytes in a patient with rheumatoid arthritis. Anterior and posterior (A,B) whole body images of a patient 2 hours after infusion of monocytes labeled with 283 MBq ^99m^Tc-HMPAO. Transient pulmonary accumulation occurs, with an equivalently increased uptake in liver, spleen and bone marrow (A,B). A reference source is placed just below the knees. Panel C and D show the same images but with masking of the pulmonary, bladder and source signal. Increased articular uptake is observed in di-arthrodial joints as the shoulders, elbows, knees and small hand joints.

Migration of labeled monocytes was visualized using scintigraphy. The majority of monocytes was initially trapped in the lungs, followed by redistribution in liver, spleen and bone marrow (Figure 1), following the pattern of labeled leukocytes [19]. As expected, renal activity with visualization of the urinary bladder was seen in all patients. Furthermore, physiological bowel uptake could be detected from one hour post infusion. Significant uptake of radioactivity in stomach and/or thyroid was not observed. In 2 patients whole-body imaging was feasible up to 20 hours post infusion.

**Figure 2:**
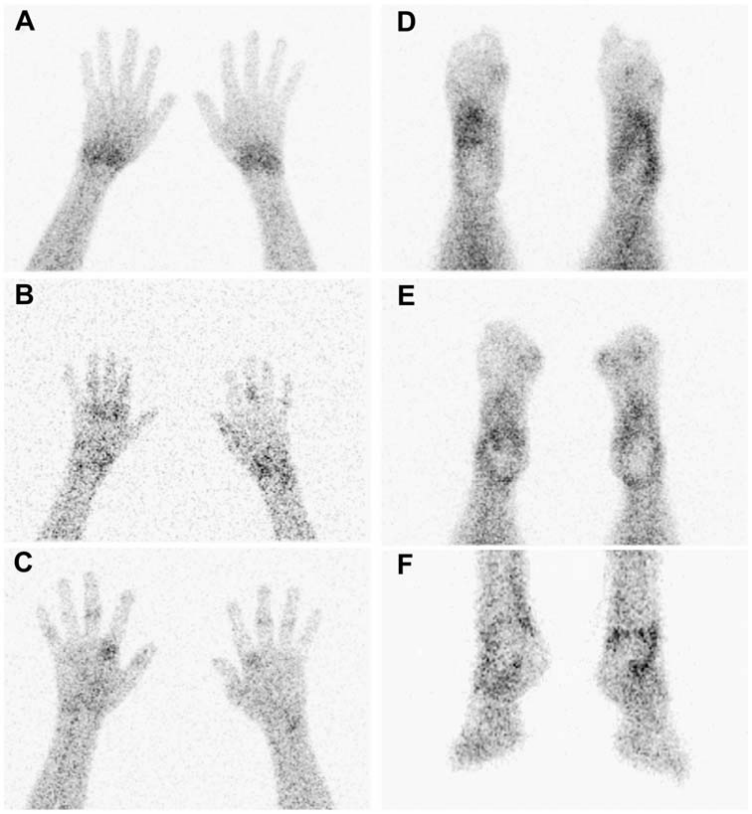
Scintigraphic detail images of hands and feet of labeled autologous monocytes in a patient with rheumatoid arthritis. Scintigraphic detail images of hands in palmar position (A–C) and feet in plantar position (D,E) and anterior position (F) of RA patients 2 h after infusion of monocytes labeled with 99mTc-HMPAO. Images of the hands show increased uptake in the wrists, MCP and IP joints (A–C). Images of the feet show increased uptake of the ankle, tarsus, MTP and IP joints (D–F).

**Figure 3:**
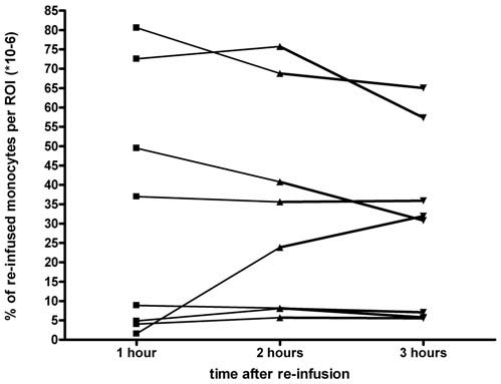
Percentage of re-infused monocytes in a joint in time. Detailed signal intensity calculation of percentage of re-infused monocytes in a selected joint of the individual patients in time after infusion. A stable presence of monocytes in the joints is visible.

Small but distinct uptake was found in the joints of all patients, with a mean of 9 (range 1–25) positive joints (Figures 1 and 2). There was an increased signal in di-arthrodial joints in all patients at all time points, with a maximal signal at one hour post re-infusion (Figure 3). We calculated that a median of 4827 (interquartile range [IQR] 2094–8370) labeled monocytes migrated into the biopsied joints that were analyzed in more detail, representing 3.4×10^−3^ (0.95−5.1×10^−3^) % of re-infused monocytes. The results were confirmed after 2 weeks, when the scans were repeated: there was no change in clinical parameters, the number of joints with increased signal on the scintigraphic images, and joint signal intensity. Using these numbers, an estimate was made about the extent of monocyte influx into the biopsied joints. The median monocyte concentration in peripheral blood was 1.9×10^9^ (1.6−2.3×10^9^) at the time of blood withdrawal. Assuming an average blood volume of 5 liters, a median of 323.000 (272.000–1.150.000) monocytes entered the biopsied joints (3.4×10^−3^ % of total circulating monocytes).

Since macrophages are the dominant synovial inflammatory cell population and the extent of their tissue infiltration correlates with disease activity [1]–[3], [20], we compared scintigraphic signal intensity with synovial macrophage infiltration and disease activity parameters. Synovial macrophage infiltration was assessed by immunohistochemical staining of arthroscopic biopsies from selected joints. These biopsy samples had been obtained one day after the second scintigraphy. Six out of 8 synovial biopsies passed synovial tissue quality control. The number of CD163 positive macrophages in the synovium correlated significantly with the number of scintigraphy positive joints one, two and three hours after re-infusion (r = 0.89, P = 0.019; r = 0.89, P = 0.019; r = 0.94, P<0.01, respectively). Furthermore, there was a significant correlation between the number of CD163 positive macrophages and the percentage of monocytes shown by scintigraphy two hours after re-infusion (r = 0.89, *P* = 0.019). The other immunohistologic markers were not significantly correlated with scintigraphic data (data not shown). Subsequently, the relationship between scintigraphic signal and disease activity parameters was analyzed. Of interest, the swollen joint count correlated significantly with the percentage of monocytes in the biopsied joints selected for detailed quantification on the images that were taken one and two hours after re-infusion (day 1: r = 0.97 and r = 0.73, respectively; P<0.01, day 14: r = 0.78 and r = 0.90; P<0.01). There was also a positive correlation between the number of swollen joints and the number of positive joints shown by scintigraphic scans at day 14 (r = 0.81, r = 0.76 and r = 0.81; all P<0.01, at respectively 1, 2 and 3 hours after infusion).

## Discussion

In the present study we used a recently developed procedure, that visualizes the migratory behavior of monocytes [18], [19], to test the hypothesis that synovial inflammation in RA is maintained by influx of monocytes into the synovial compartment. The results suggest that while there is indeed a continuous influx of circulating monocytes into the synovial compartment, their numbers are small, indicating that only a relatively small fraction of synovial macrophages is replaced per day.

We found that a median of 3.4×10^−3^ (0.95−5.1×10^−3^) % of labelled monocytes entered the synovial compartment after re-infusion. Since we found no indication that the monocytes were activated by the labelling or isolation procedures, this suggests that similar percentages of unlabeled circulating monocytes enter the synovial compartment, being a median of 323.000 cells. Since the number of synovial macrophages in large inflamed joints exceeds such numbers considerably, these data indicate that in RA patients the rate of synovial macrophage renewal by circulating monocytes is slow.

It was recently shown that a range of anti-rheumatic treatments induces a significant decrease in synovial macrophages associated with clinical improvement within hours to weeks after initiation of therapy [5], [6]. It was hypothesized that these treatments ultimately affect migration of monocytes and/or retention of macrophages [21]. In studies on infliximab, an effective biological treatment for RA which blocks TNFα [22], a marked decrease in synovial macrophage numbers occurred already 24 hours after initiation of treatment, which could not be explained by induction of apoptosis [8]–[11]. The results of the current study suggest that this effect may mostly explained by an effect of TNFα blockade on macrophage retention. Of interest, a decrease in VCAM-1 and ICAM was found shortly after infliximab treatment [8], [10]. While these are important molecules involved in adhesion of monocytes to the vascular endothelium, they are in RA patients also abundantly expressed by cells in the intimal lining layer and the synovial sublining, where they play a pivotal role in inflammatory cell retention and survival [23].

Thus, the data indicate that in line with animal models on chronic inflammatory lesions, macrophage renewal by monocytes is slow in chronic synovitis [12]–[15]. Together with the previous studies showing a rapid decrease in cellularity after successful antirheumatic therapy (even in the absence of apoptosis), these data support the notion that therapeutic strategies aimed at interfering with retention of inflammatory cells at the site of inflammation might be capable of inducing clinical improvement in immune-mediated inflammatory disease. Accordingly, recent studies have shown benefit of interfering with adhesion molecules: anti-function associated antigen (LFA)-1 antibody (efalizumab) treatment in psoriasis and anti-α4β1-integrin (VLA-4) antibody (natalizumab) treatment in multiple sclerosis [24], [25].

In conclusion, we developed monocyte scintigraphy, which allowed us to demonstrate the dynamic influx of monocytes into the synovial compartment of RA patients. This approach provides insight into the pathogenesis of this immune-mediated inflammatory disease and supports the notion that blocking only the influx of inflammatory cells may be insufficient to induce clinical improvement.

## Materials and Methods

### Patients

Patients fulfilling the American College of Rheumatology (ACR) 1987 revised classification criteria for RA [26] were included into the study. All patients had active RA, as defined by a disease activity score evaluated in 28 joints (DAS28) >3.2 [27]. Patients were on stable disease-modifying antirheumatic drug (DMARD) treatment at inclusion.

### Ethics Statement

Approval was granted by the Medical Ethics Committee of the Academic Medical Center/University of Amsterdam (AMC). Each patient gave written informed consent prior to participation.

### Isolation of Monocytes

Hundred milliliters of peripheral blood was taken from each patient. CD14+ monocytes were isolated using a positive selection procedure with magnetic-activated cell sorting according to the manufacturer's protocol (MACS®Miltenyi Biotech, Bergisch Gladbach, Germany). After selection, the percentage of CD14, CD3, and CD66 positive cells was determined by fluorescence-activated cell sorting (FACS) analysis. The CD14+ enriched cells were resuspended in 10 ml buffer containing 0.9% (w/v) NaCl, 20% (w/v) human serum albumin (Sanquin Blood Supply Foundation division of Plasma Products, Amsterdam, the Netherlands) and 3.8% (w/v) TNC (NVI, Bilthoven, the Netherlands) for labeling.

### Radiopharmaceuticals

Exametazime (Ceretec™, RVG16226) was supplied as a ready-for-labeling kit (GE Healthcare B.V., Amersham, Cygne Centre, Eindhoven, the Netherlands). ^99m^Tc-pertechnetate was obtained from a ^99^Mo-carrying Ultratechnekow® FM generator (DRN 4329, Tyco Healthcare, Mallinckrodt Medical, Petten, the Netherlands) and was eluted in accordance with the instructions of the manufacturer. Radiochemical purity control (RPC) assays were done by means of chromatography on ITLC-SG strips, using a mobile phase of 0.9% sodium chloride (NaCl) [28]. Radiolabeling of cells was performed as described earlier [18]. Briefly, the cells were centrifuged and freshly prepared ^99m^Tc-HMPAO of very high specific activity in a low volume was added to the monocyte cell pellet. After incubation the excess of ^99m^Tc-HMPAO was diluted and subsequently removed from the cell pellet after centrifugation. The labeled monocytes were resuspended in 0.9% NaCl and re-infused into the same patient.

### Scintigraphy

An average of 20×10^6^ monocytes labeled with 200 MBq ^99m^Tc-HMPAO was injected intravenously within 15 minutes after radiolabeling. Whole body imaging was performed at 15 minutes and 1, 2, 3, and 20 hours post infusion using a dual head gammacamera (140 keV, window 15%, 256×1024 matrix, 10 cm/min) fitted with low energy all purpose collimators (Siemens Ecam). Detail images of the hands (palmar) and feet (plantar) were acquired in a 256×256 matrix for 5 minutes. This procedure was repeated two weeks after the baseline scintigraphy.

### Signal Calculations

The scintigraphic scans were analyzed for signal intensity in joints and other tissues. The number of positive joints and the exact signal intensity of the biopsied joint was selected for more detailed quantification. The signal intensity was calculated in counts per region of interest, subtracting the background signal from the joint signal. A correction was made for the number of re-infused monocytes and the injected dose, using a standard dose source, leading to a deduction of the percentage of re-infused monocytes per ROI.

### In Vitro Assays to Determine the Influence of Isolation and Labeling on Monocyte Migratory Function and Activation

Monocytes were isolated from whole blood of subjects and migratory function was assessed by in vitro chemotaxis assay comparing radioactively labeled and non-labeled cells. Briefly, chemotaxis assay was done using 24-well chemotaxis plates (Corning Costar, Corning, NY) with inserts containing pre-grown ECV304 cells. Purified monocytes were re-suspended in 600 ul of pre-warmed RPMI medium containing 3% fetal calf serum (GIBCO, BRL) and added to the upper chamber of the transwell plate. Chemotaxis was performed for 2 hours at 37°C against various concentrations of recombinant MCP-1 (R&D Systems, Minneapolis, MN). Cell migration rate was quantified by flow cytometry for non-labeled monocytes and scintillation gamma counter for labeled monocytes. Comparison of migrating cell percentage for non-labeled and Tc- HMPAO labeled monocytes did not demonstrate an impairment of monocyte migratory capacity in the in vitro chemotaxis assay (data not shown).

Monocyte activation was tested by assessing CD62L expression by flow cytometry on monocytes in unmanipulated whole blood and after the bead isolation procedure using CD62L-PE conjugated antibody (BD Biosciences). Antibody concentrations were used according the manufacturer's protocol. Antibody staining was performed using 50 µl of whole blood. Blood cells were incubated with antibodies for 15 min and washed twice with PBS containing 1% bovine serum albumin (Sigma-Aldrich, MO). Red cells were lysed by washing cells twice with BD FACS™lysis solution (BD Biosciences, CA). Purified monocytes were resuspended in PBS containing 1% bovine serum albumin (Sigma-Aldrich, MO), incubated with antibody and fixed with FACS™lysis solution (BD Biosciences, CA). Samples were analyzed by flow cytometry using a FACS Calibur (Becton Dickinson, NJ). Frequency of CD62L expression on monocytes did not change after the bead isolation procedure (data not shown).

### In Vivo Assessment of Complement Activation after Re-Infusion

To exclude the possibility that re-infusion of labeled monocytes induced complement activation, complement activation products were measured in the serum before and one hour after re-infusion. Activation of C3 (C3b/c) and C4 (C4b/c) was assessed with an ELISA as described before in detail [29]. In brief, monoclonal antibodies recognizing neo-epitopes on activated C3 and C4 were used as capturing antibodies. Biotinylated polyclonal rabbit anti-human C3 and polyclonal sheep anti-human C4 antibodies were used as detecting antibodies.

### Arthroscopy and Synovial Biopsy

The day after the second set of scans, all patients underwent a mini-arthroscopy under local anesthesia from an actively inflamed knee, ankle or wrist, to obtain synovial tissue samples [30]. Biopsies were taken with a 2.3-mm grasping forceps (Storz, Tuttlingen, Germany) from 6 or more sites within the joint to minimize sampling error. The tissue samples were snap frozen en bloc in Tissue Tek OCT (Miles, Elkhart, IN) after collection. Frozen blocks were stored in liquid nitrogen until sectioning. Sections of 5 µm were cut using a cryostat and mounted on Star Frost adhesive glass slides (Knittelgläser, Braunschweig, Germany). Sealed slides were stored at −80°C until immunohistochemical staining was performed.

### Immunohistochemical Analysis

Synovial tissue sections were stained using the following monoclonal antibodies to analyze the cell infiltrate: anti-CD55 (67:Serotec, Oxford, UK) to detect fibroblast-like synoviocytes (FLS), anti-CD68 (EBM11: DAKO, Glostrup, Denmark) to detect macrophages and anti-CD3 (SK7, Becton Dickinson, San Jose, CA) for T-cells as described previously [31]. The scavenger receptor CD163 (Ber-MAC3; DAKO) was stained to detect alternatively activated tissue macrophages. Staining of cellular markers was performed using a three-step immunoperoxidase method [32]. For control sections the primary antibody was omitted or irrelevant immunoglobulins were applied. Tissue quality was assessed by analyzing the presence of an intimal lining layer.

### Digital Image Analysis

All sections were analyzed at random by trained technicians who were blinded for clinical and scintigraphic data. The analysis was done by computer-assisted image analysis as previously described in detail (323). In short, images were acquired and analyzed using a Syndia algorithm on a Qwin-based analysis system (Leica, Cambridge, UK). For all markers 18 high-power fields were analyzed. Positive staining of cellular markers was expressed as positive cells per mm^2^ (counts/mm^2^). CD68+ macrophages were analyzed separately for the intimal lining layer and the synovial sublining.

### Statistics

Associations between joint signal parameters and swollen joint count, tender joint count, DAS28, ESR, CRP and immunohistochemical markers were expressed by Spearman's correlation coefficients. The changes in joint signal intensity and clinical parameters after two weeks were tested with the Wilcoxon signed ranks test for paired non-parametric data.
